# Arsenic trioxide alters the differentiation of mouse embryonic stem cell into cardiomyocytes

**DOI:** 10.1038/srep14993

**Published:** 2015-10-08

**Authors:** Paola Rebuzzini, Elisa Cebral, Lorenzo Fassina, Carlo Alberto Redi, Maurizio Zuccotti, Silvia Garagna

**Affiliations:** 1 Laboratorio di Biologia dello Sviluppo, Dipartimento di Biologia e Biotecnologie ‘Lazzaro Spallanzani’, Università degli Studi di Pavia, Pavia, Italy; 2 Laboratorio de Reproducción y Fisiopatología Materno-Embrionaria, Instituto de Fisiología, Biología Molecular y Neurociencias, Departamento de Biodiversidad y Biología Experimental, Facultad de Ciencias Exactas y Naturales, Universidad de Buenos Aires, Buenos Aires, Argentina; 3 Dipartimento di Ingegneria Industriale e dell’Informazione, Università degli Studi di Pavia, Pavia, Italy; 4 Center for Health Technologies (CHT), Via Ferrata 1, Università of Pavia, Italy; 5 Centro Ricerche di Medicina Rigenerativa, Fondazione IRCCS Policlinico San Matteo, Pavia, Italy; 6 Unita’ di Anatomia, Istologia ed Embriologia, Dipartimento di Scienze Biomediche, Biotecnologiche e Traslazionali, Università degli Studi di Parma, Parma, Italy

## Abstract

Chronic arsenic exposure is associated with increased morbidity and mortality for cardiovascular diseases. Arsenic increases myocardial infarction mortality in young adulthood, suggesting that exposure during foetal life correlates with cardiac alterations emerging later. Here, we investigated the mechanisms of arsenic trioxide (ATO) cardiomyocytes disruption during their differentiation from mouse embryonic stem cells. Throughout 15 days of differentiation in the presence of ATO (0.1, 0.5, 1.0 μM) we analysed: the expression of i) marker genes of mesoderm (day 4), myofibrillogenic commitment (day 7) and post-natal-like cardiomyocytes (day 15); ii) sarcomeric proteins and their organisation; iii) Connexin 43 and iv) the kinematics contractile properties of syncytia. The higher the dose used, the earlier the stage of differentiation affected (mesoderm commitment, 1.0 μM). At 0.5 or 1.0 μM the expression of cardiomyocyte marker genes is altered. Even at 0.1 μM, ATO leads to reduction and skewed ratio of sarcomeric proteins and to a rarefied distribution of Connexin 43 cardiac junctions. These alterations contribute to the dysruption of the sarcomere and syncytium organisation and to the impairment of kinematic parameters of cardiomyocyte function. This study contributes insights into the mechanistic comprehension of cardiac diseases caused by *in utero* arsenic exposure.

Arsenic, a natural element present on the earth’s crust and a product of industrial activities, is a widely diffused environmental toxicant. Contamination of groundwater with arsenic has been recognised as a massive public health hazard^1^,^2^ and an estimated >140 million people worldwide^3^ are chronically exposed at concentrations exceeding the WHO limit (10 μg/L)^4^.

The effects of arsenic on human health include: neurological disorders, cancer, gastrointestinal disturbances, dermal, liver, renal, fertility and cardiovascular diseases^5^,^6^,^7^,^8^,^9^,^10^. Epidemiological studies have evidenced the strong association between chronic arsenic exposure and increased morbidity and mortality for cardiovascular diseases^6^,^11^. Even low levels of arsenic exposure have been related to increased risks of hypertension^12^,^13^, carotid atherosclerosis^14^, diseases of arteries, arterioles and capillaries^15^,^16^ and ischemic heart disease^17^,^18^.

As shown in a recent systematic review and meta-analysis study, environmental exposure *in utero* to arsenic compounds causes adverse pregnancy outcomes such as increased risk of spontaneous abortion, stillbirth, reduction in birth weight, moderate risk of neonatal and infant mortality^19^. Also, it has a major effect on mortality in young adulthood due to myocardial infarction^11^,^20^, suggesting that exposure during foetal life may induce cardiac alterations that will emerge later.

Arsenic trioxide (ATO), an environmental contaminant listed on the Agency for Toxic Substances and Disease Registry priority list of hazardous substances (#225; http://www.atsdr.cdc.gov/SPL/), is associated with cardiac toxicity, inducing cardiac arrhythmia and high rate of apoptosis in cardiomyocytes, as a consequence of the production of reactive oxygen species and the induction of calcium overload^21^. These toxic effects have also been reported in patients treated with ATO in combination with retinoic acid for the treatment of haematological malignancies^22^,^23^ and solid tumors^24^. Whilst the literature provides numerous evidences of the toxic effects of ATO on the cardiovascular system, as of today, the knowledge of its impact during the process of cardiomyocyte differentiation is meagre.

*In vitro* studies on a rat cardiomyocyte line (H9c2), derived from foetal heart, demonstrated that ATO (2–10 μM for 24 h) induces apoptosis in a concentration-dependent manner^25^. Also, when exposed to 3 μM ATO for 24 h, these cells showed a reduced ability to metabolize and excrete arsenic compared to other non-foetal rat-derived cell lines^26^. In a further study, mouse embryonic stem cells (mESCs), continuously treated with 0.7–1.3 μM ATO for 10 days during their differentiation to cardiomyocytes, did not show beating capacity when analysed with the embryonic stem cell test (EST)^27^; and, when treated with 0.5–1.0 μM monomethylarsonic acid (a methylated arsenic metabolite) for 1–3 days ceased proliferation and cardiomyocyte differentiation^28^. Altogether, these studies suggest that ATO and its metabolites exert adverse effects during cardiomyocyte differentiation.

Although epidemiological studies suggest a link between *in utero* exposure to ATO and the risk to develop cardiac pathologies later in life, up to date there are no studies that analyse the effects of ATO neither at specific steps of cardiomyocyte differentiation nor on the structural-functional features of terminally differentiated cells.

The main aim of the present study is to investigate, at a molecular and functional level, the effects that a continuous exposure to ATO has on the process leading to the formation of fully differentiated post-natal cardiomyocytes. To this end, we used mESCs as a well established *in vitro* model that recapitulates, through the formation of the three germ layers, from spheroid structures named embryoid bodies (EBs), the molecular events and the functional features of cardiomyocyte differentiation from primitive precursor cells up to highly specialised phenotypes^29^,^30^.

During the whole 15 days of differentiation, cells were continuously exposed to 0.1, 0.5 or 1.0 μM ATO and analysed for the expression of marker genes of i) early, primary myocardial-like cells (cardiac commitment; on day 4); ii) intermediate (myofibrillogenic commitment; on day 7) and iii) terminal, post-natal-like cardiomyocytes (on day 15). Also, in differentiated cardiomyocytes (day 15) we analysed the expression and organisation of sarcomeric proteins and, at a functional level, the kinematics contractile properties of syncytia.

## Results

### Effects of the vehicle NaOH on cardiomyocyte differentiation

**Figure 1:**
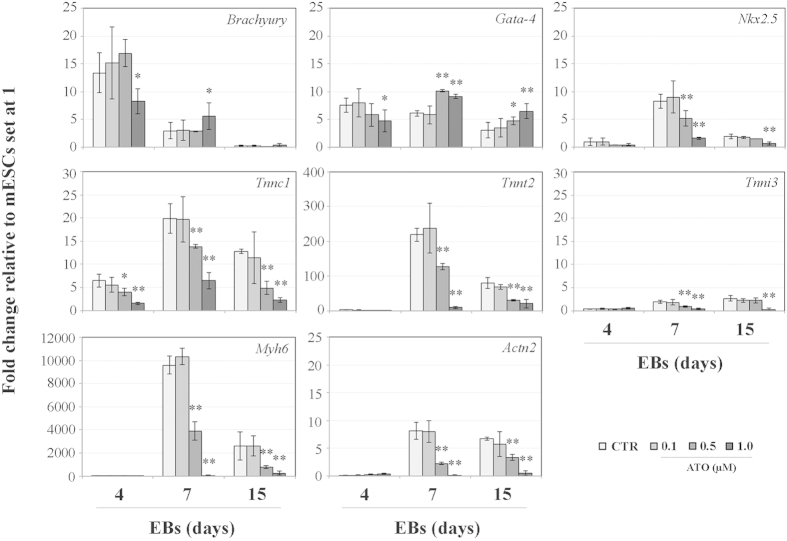
Expression profile of genes that mark mesoderm (*Brachyury*), cardiac mesoderm (*Gata-4, Nkx2.5*) and cardiac cells (*Tnnc1, Tnnt2, Tnni3, Actn2, Myh6*) in CTR and ATO-exposed EBs. Values are expressed as mean ± standard deviation. Three independent sets of this experiment were performed. ^*^p < 0.05; ^**^p < 0.001.

To verify the effects of the vehicle used to dissolve ATO on the process of differentiation, throughout the experiments described below, we cultured cells in the presence or absence of 0.01 N NaOH. Since we never observed a statistically significant difference (*p* > 0.05) when analysing the expression profile of genes that mark mesoderm, cardiac mesoderm and cardiac cells (Fig. 1S A), the kinematics contractile properties of beating syncytia (Fig. 1S B) and the immunolocalisation of sarcomeric and connexin 43 proteins (Fig. 1S C), the experiments described hereafter will report, as control sample, only the results obtained in the presence of NaOH.

### Analysis of As3MT gene expression

**Figure 2:**
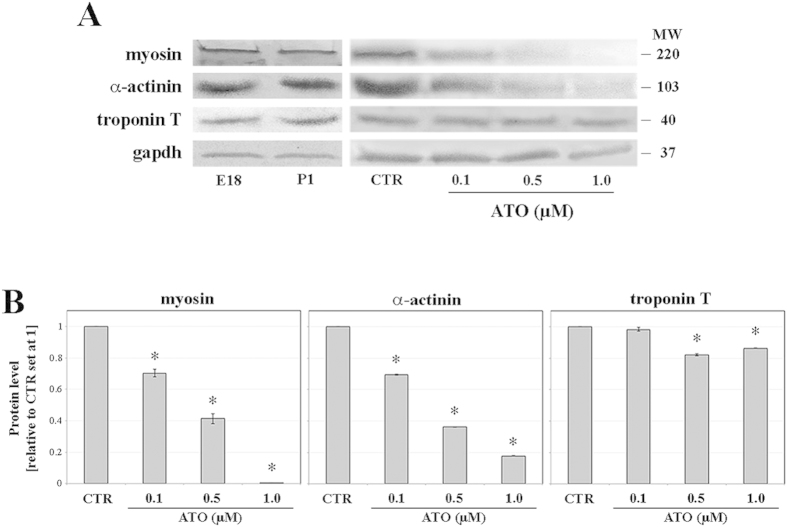
(**A**) Western blotting analysis of sarcomeric proteins of prenatal (E18) and newborn (P1) mice hearts and of beating syncytia differentiated in the absence (CTR) or in the presence of 0.1, 0.5 or 1.0 μM ATO on day 15 of the differentiation process. (**B**) Relative quantity of sarcomeric proteins. *p < 0.001

In Mammals, arsenite methyltransferase (As3MT) is a key enzyme that catalyzed the biotransformation of trivalent inorganic arsenic into mono- and dimethylated metabolites^28^,^31^,^32^. We analyzed the expression of the *As3MT* gene in undifferentiated R1 mESCs and on day 4, 7 and 15 of cultured EBs. *As3MT* was constitutively expressed in both mESCs (data not shown) and during the whole differentiation process (Fig. 2S). In the presence of 0.1 μM ATO, when compared to CTR (set at 1 for the calculation of the *n*-fold change of treated samples), the relative number of As3MT transcripts did not change significantly (*p *> 0.05) on day 4 and 7 of differentiation, whereas it showed a significant 1.7-fold change increase on day 15. In the presence of 0.5 and 1.0 μM ATO, a significant induction of *As3MT* gene expression was observed at each time point analysed.

In summary, following a continuous exposure to ATO, we observed a significant induction of *As3MT* at all ATO doses, increasing at the increasing of the concentration, suggesting that the biotransformation of arsenic occurs during the whole process of cardiomyocyte differentiation.

### Effects of ATO on the expression of marker genes of cardiomyocyte differentiation

Earlier studies have shown that the expression of cardiac-associated gene transcripts in ESC-derived cardiomyocytes is a function of the differentiation time, as in normal myocardial development^29^,^33^. Similarly, RT-PCR analyses have demonstrated that transcripts of the cardiac mesoderm *GATA-4* and *Nkx2.5* transcription factors are expressed before the mRNA of the sarcomeric proteins^34^,^35^,^36^. Here, on day 4, 7 and 15 of EBs culture, we investigated the profile of expression of specific gene markers of mesoderm (*Brachyury*), cardiac mesoderm (*Gata-4* and *Nkx2.5*) and cardiac cells (*Tnnc1*, *Tnnt2*, *Tnni3*, *Myh6* and *Actn2*) differentiation. The expression of untreated mESCs (day 0) was used as reference value (set at 1) for the calculation of the *n*-fold change during the differentiation process. Figure 1 describes the details of the profile of expression of these genes. The presence of 0.1 μM ATO in the culture medium did not alter significantly (*p *> 0.05) neither the profiles nor the quantitative expression of all the genes analysed, which were similar to those determined for CTR samples. Treatment with 0.5 μM ATO did not alter the expression of *Brachyury*, but increased *Gata-4* (day 7) and decreased *Nkx2.5*, *Tnnt2*, *Tnni3*, *Myh6* and *Actn2* (day 7), whereas *Tnnc1* decreased earlier (day 4). All genes maintained an increasing or decreasing profile also on day 15, with the exception of *Nkx2.5* and *Tnni3* that attained levels of expression similar to those measured in CTR samples. Cells cultured in the presence of 1.0 μM ATO showed significant variations in the number of transcripts of all the marker genes analysed, with the exception of *Brachyury* on day 15 and *Nkx2.5*, *Tnnt2*, *Tnni3*, *Myh6*, *Actn2* on day 4.

In summary, whilst we did not detect differences in the profile of expression of genes that mark the progression of mESCs differentiation towards cardiomyocytes when comparing CTR *vs*. 0.1 μM ATO-treated cells, significant variations were observed at higher concentrations, suggesting an alteration of both the myofibrillogenic commitment (day 7) and the terminal cardiomyocyte differentiation (day 15). Furthermore, at 1.0 μM ATO concentration, a decreased expression of *Brachyury* indicates that also the mesoderm commitment (day 4) might be altered.

Since these results showed an alteration of the transcription profile of key genes whose proteins are critical components of the sarcomere, next we analysed by Western blotting the quantitative profile of expression of myosin, cardiac α-actinin and troponin T proteins and investigated by immunofluorescence cardiac α-actinin, troponin T and Cx43, the latter a component of the gap junctions between cardiomyocytes.

### Quantification of sarcomeric proteins

When compared to CTR, at 0.1 μM ATO concentration, myosin and α-actinin proteins were reduced by 30%, whilst troponin T expression remained unaltered (Fig. 2). At 0.5 or 1.0 μM ATO, all sarcomeric proteins were significantly reduced. At both doses, troponin T was reduced by about 20%, α-actinin decreased by 65% at 0.5 μM and by 80% at 1.0 μM. Myosin diminished by 60% at 0.5 μM and it was almost undetectable at 1.0 μM (Fig. 2B).

The quantitative ratio 1:1.5:3 of troponin T, myosin and α-actinin, respectively, observed in our CTR samples is consistent with that of prenatal (E18) or newborn (P1) cardiomyocytes (Fig. 2A; Table 1). In cardiomyocytes differentiated in the presence of ATO, abnormal stoichiometry of sarcomeric proteins was observed with a major reduction of the ratio at increasing doses (Table 1). This reduction does not reflect a decreased efficiency of the differentiation process since the frequency of differentiated cardiomyocytes (α-actinin-positive) does not differ significantly (*p *> 0.05) when comparing CTR (40.5% ± 3.2) *vs*. 0.1 μM (38.5% ± 3.1), 0.5 μM (39.4% ± 2.7) or 1.0 μM (37.4% ± 4.2) samples. Thus, we hypothesised that the skewed protein ratio may interfere with the correct organisation of the sarcomere.

### Sarcomeric organization of cardiomyocytes

On day 15 of differentiation, we evaluated, by immunofluorescence, the localisation of the α-actinin and troponin T sarcomeric proteins and of Cx43 which identifies the presence of gap junctions between cardiomyocytes.

**Figure 3:**
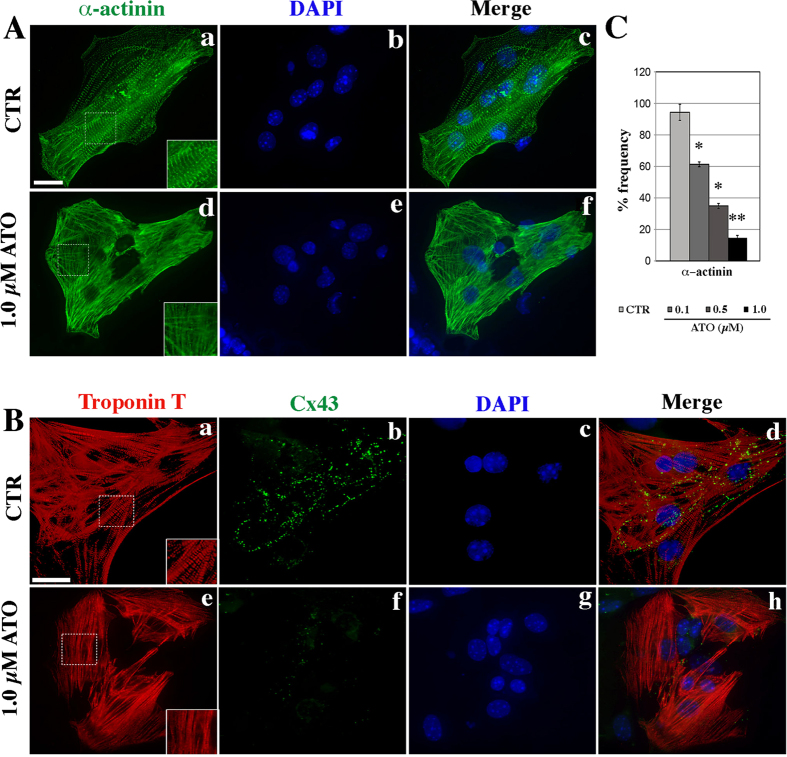
Immunofluorescence localisation of (A) cardiac α-actinin and of (B) cardiac troponin T (red) and of Connexin 43 (green) proteins in cardiomyocytes derived CTR or from 1.0 μM ATO-treated mESCs at day 15 of differentiation. For each antibody, about 400 cells were analysed. Bar, 20 μm. (**C**) Frequency of cardiomyocytes with correct sarcomeric organization. Immunofluorescence was performed on partially disaggregated syncytia; the frequency was calculated counting the number of nuclei. The immunofluorescence pattern was homogeneous per group of cells, i.e. all cells presented either a disorganised or a correct striated pattern. ^*^p < 0.01; ^**^p < 0.001.

On CTR cardiomyocytes, α-actinin and troponin T displayed the sarcomere-specific striated pattern (Fig. 3A,B). On the contrary, cardiomyocytes cultured with either 0.1, 0.5 or 1.0 μM ATO presented disorganized and disoriented sarcomeres with an evident alteration of the myofibrillar components and absence of the striated configuration for both α-actinin (Fig. 3Ad) and troponin T (Fig. 3Be) proteins. The frequency of cardiomyocytes displaying a correct sarcomere organisation decreased with increasing ATO doses (Fig. 3C), from 61.3% (*p* < 0.01) (0.1 μM ATO) to 14.7% (*p* < 0.001) (1.0 μM ATO) (Fig. 3C).

Double immunostaining on CTR cardiomyocytes exhibited correct sarcomere organisation (Fig. 3Ba) and Cx43 foci (Fig. 3Bb) densely distributed along straight lines. Instead, treated cardiomyocytes, in addition to an altered sarcomere organisation (Fig. 3Be), showed few and dispersed Cx43 foci (Fig. 3Bf). Despite this altered sarcomere organisation, treated cardiomyocytes exhibited a contractile activity. Next, we investigated further into their contractile properties.

### Kinematics contractile properties of cardiomyocyte syncytia

An inherent feature of ESC-derived cardiomyocytes is the acquisition of spontaneous contractile activity^29^,^37^,^38^. Thus, we monitored the differentiation of cardiomyocyte syncytia from day 7 to 15 to determine the timing of acquisition of their beating capacity.

A total of 437 CTR, 451 0.1 μM and 444 0.5 μM ATO-treated EBs were plated in three independent experiments. All EBs of these three groups showed clear contractile activity beginning from day 7 of differentiation and maintained it until the end of the culture period. Instead, only 19.2% EBs (84 out of 437) cultured in 1.0 μM ATO showed beating activity beginning on day 12 of differentiation (5 days later compared to CTR), a frequency maintained until day 15. A further specificity of syncytia differentiated in 1.0 μM ATO was the presence of one or few small beating foci compared to CTR or EBs treated at lower concentrations, in which contraction of the whole cell mass was observed (see videos supplementary materials).

**Figure 4:**
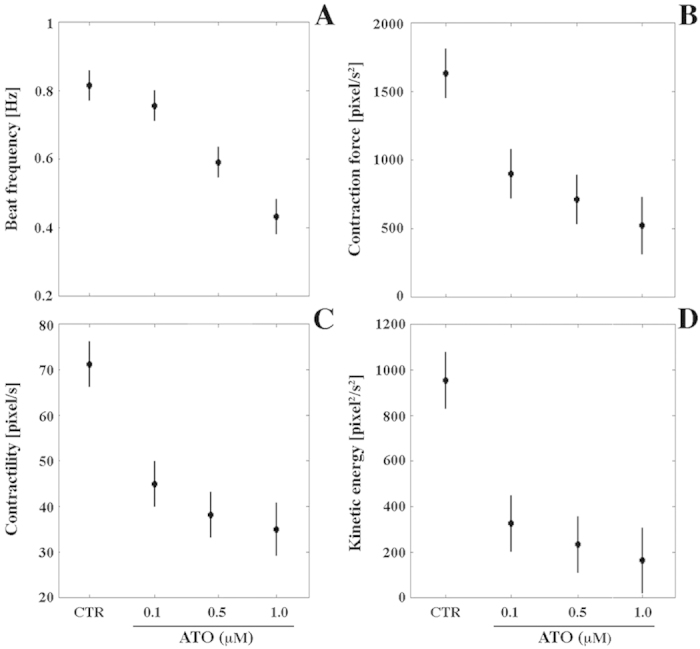
Contractile properties of beating syncytia differentiated from CTR or in the presence of 0.1, 0.5 or 1.0 μM ATO on day 15 of the differentiation process. (**A**) Beat frequency [Hz]; (**B**) Contraction force [pixel/s^2^]; (**C**) Contractility [pixel/s]; (**D**) Kinetic energy [pixel2/s^2^]. Horizontal bars represent the 95% confidence intervals for the differences between means according to the Least Significant Difference statistical test.

Next, to evaluate the contractile properties of beating syncytia, we measured their kinematics and dynamics features on recorded AVI videos. The chronotropic (beat frequency [Hz]), inotropic (contraction force [pixel/s^2^] and contractility [pixel/s]) and ergotropic (consumption of ATP for kinetic energy [pixel2/s^2^]) effects were mathematically calculated from the movement of the beating syncytia^39^ (Fig. 4). This analysis showed that cardiomyocytes differentiated in the presence of 0.1 μM ATO, whilst maintaining an unaltered beat frequency (Fig. 4A) (*p* > 0.05), displayed a significant reduction (*p* < 0.05) of contractility, contraction force and kinetic energy (1.5-, 3-, and 1.7-fold, respectively) (Fig. 4B–D). A more detrimental effect was recorded for syncytia differentiated in 0.5 or 1.0 μM ATO compared to CTR (Fig. 4)  (*p* < 0.05). Specifically, we observed a 4.3-, 2.3-, 1.4-, 1.9-fold decrease of beat frequency, contraction force, contractility and kinetic energy, respectively, when cells were cultivated in the presence of 0.5 μM ATO; and a 5.3-, 4.5-, 2-, 3.2-fold decrease of beat frequency, contraction force, contractility and kinetic energy, respectively, when cells were cultured in the presence of 1.0 μM ATO.

## Discussion

Environmental exposure to arsenic during prenatal life increases the appearance of cardiac disease after birth^11^,^20^. With the aim of investigating the effects that arsenic exerts during cardiomyocyte differentiation, we used an *in vitro* cell system that recapitulates the molecular events and the functional features of cardiomyocyte differentiation from mesoderm to specialised cardiac cells^29^,^40^. To the best of our knowledge, this study is the first to demonstrate specific effects of ATO throughout the whole 15 days of *in vitro* differentiation of mESCs into cardiomyocytes, resulting in the disruption of this developmental process. Throughout culture, cells were continuously exposed to 0.1, 0.5 or 1.0 μM ATO, doses in the range of inorganic arsenic determined in contaminated water^3^,^41^. Our results describe a dose-dependent impact on the cardiomyocyte differentiation process; in fact, at increasing ATO doses, the frequency of cardiomyocytes with altered sarcomere organisation augmented. Furthermore, the higher the dose used, the earlier was the stage of differentiation affected. With 0.1 μM ATO, alterations are observed only at the very end of the differentiation process, with cardiomyocytes displaying a decreased quantity and a skewed ratio of sarcomeric proteins, together with disorganized sarcomeres, reduced Cx43-positive gap junctions and contractility, contraction force and kinetic energy. When ATO is increased to 0.5 μM, its effects are observed earlier during the differentiation process. In addition to the effects described with the 0.1 μM dose, also the myofibrillar commitment is clearly affected and, at a further increase to 1.0 μM ATO, alterations are observed as early as mesoderm formation. Also, the acquisition of the beating capacity is acquired by only a fifth of EBs, with a five-day delay, i.e., on day 12 of differentiation. These latter results mark the importance of analysing the process until the end of the 15 days differentiation period, as we observed a retardation in the acquisition of the contraction capacity depending on the ATO dose. To this regard, a previous study, where mESCs were continuously treated with 0.7–1.3 μM ATO, reported the absence of the acquisition of the spontaneous cardiomyocyte contraction activity, when the differentiation process lasted only for 10 days^27^.

Although during treatment with the lowest ATO dose we could not detect variations to the expression of the transcript markers employed, the end-point of the differentiation process was a cardiomyocyte with affected morpho-functional features, similar to those reported for higher doses. Perhaps, this might be explained with effects occurring at the post-transcriptional level, i.e., lower sarcomere proteins expression and their skewed ratio.

Instead, at higher ATO doses, the detrimental effect is observed even on transcription and, at 1.0 μM ATO, already at the time of mesoderm commitment, when the expression of *Brachyury* is lower than that of CTR. As for *Gata-4* and *Nkx2.5*, at the time of myofibrillar commitment, we observed an altered up- and down-regulated expression, respectively, that does not reflect their known mutual positive regulation^42^,^43^,^44^. We surmise that, in ATO-treated samples, the low *Nkx2.5* transcripts level might be a consequence of the interruption of the regulation that *Gata-4* exerts on *Nkx2.5* and/or the dysregulation of *Nkx2.5* autoregulation^44^. The increased level of *Gata-4* observed may be explained through the synergic action of *Nkx2.5* (despite its lower expression level) and the delayed down-regulation of *Brachyury*, which, by inducing *Mesp1*, positively regulates *Gata-4* transcription^45^. The effect at higher doses on the level of gene expression is further evidenced by the alteration of *Tnnc1*, *Tnnt2*, *Tnni3*, *Myh6* and *Actn2*. Similarly, at increasing (0.1, 0.5, or 1.0 μM) ATO doses the expression of myogenic genes (*Myf5*, *myoD* and *myogenin*) is reduced in mouse embryonal carcinoma P19 cells when induced to differentiate into myoblasts^46^. As a consequence of these transcriptional changes, the repercussion on the differentiation process is manifested with an inhibition of myogenic differentiation^47^, a reduced capacity to form myotubes^46^ or a delayed EBs beating capacity (our work). The smaller size of beating foci and their retarded contraction activity suggest a slowdown of the differentiation process, as previously reported for mouse C2C12 myoblasts^48^ and for H9c2 cells^49^ exposed to 20 nM or 0.1–1.0 μM sodium arsenite, respectively.

Besides observing alterations to the transcription of cardiomyocyte-specific genes, we also describe, for the first time, that, even at the lowest dose used, ATO leads to a quantitative reduction and to a skewed ratio of sarcomeric proteins. This skewed ratio might explain the dysruption of the sarcomere organisation and the alteration of the kinematics and dynamics properties of the beating syncytia. In fact, the correct ratio of sarcomeric proteins is crucial to the proper formation of the sarcomere and thus to the contraction and relaxation activity of cardiac cells^50^.

Another effect that we recorded is a more rarefied distribution of the cardiac junctions marked by Cx43, a diminution that has also been observed in human aortic endothelial cells treated with ATO^51^. The reduction of Cx43-positive cardiac junctions may interfere with the cell-to-cell communication^51^ and be causal, together with an altered sarcomere organisation, of the impaired kinematic parameters, as already reported for cardiomyocytes of Cx43 knock-out mice that show an altered beating frequency^52^. A key point of investigation will be the analysis of the functionality of the ion-channels, crucial actors in the transmission of the beating stimulus within the syncytium and whose specific genes have already been shown to be target of ATO exposure in human adult cardiomyocytes^53^.

Whilst the alterations that we reported in the present study explain the impaired functionality of the differentiated cardiomyocytes, other effects of ATO likely concur to the phenotype observed. A crucial point of ATO toxicity is its biotransformation into mono- and dimethylated metabolites^31^,^32^ catalyzed by As3MT, an enzyme that we have shown to be constitutively expressed in the R1 mESC line and during the whole process of cardiomyocyte differentiation. Although the pathways involved in ATO metabolism remain debated^32^, we observed a significant induction of *As3MT* at all ATO doses, increasing with the increase of the concentration, suggesting that the methylation of arsenic occurs during the whole differentiation process. Interestingly, among the arsenic metabolites produced, the monomethylarsonous acid was found to exert adverse effects inhibiting ESCs differentiation into cardiomyocytes^28^. Future studies will have to focus on the identification and quantification of the metabolites produced in our experimental setup and their action upon this differentiation process.

## Conclusions

Our study shows that the presence of ATO throughout differentiation leads to the formation of morphologically and functionally altered cardiomyocytes and endorses the use of ESCs as an *in vitro* model to study the effects that ATO exerts at each step of differentiation. Also, this work highlights the importance of monitoring the whole process throughout, since the molecule has adverse effects at different time points. As a whole, our results may contribute insights into the mechanistic comprehension of specific human cardiac diseases caused by *in utero* arsenic exposure^54^.

## Methods

### Cell lines

R1 mouse embryonic stem cell line (kindly provided by Dr. Nagy from Samuel Lunenfeld Research Institute, Mount Sinai Hospital, Toronto, Ontario, Canada) and STO cell line were cultivated as previously described^55^.

### Differentiation of mESCs into cardiomyocytes

mESCs were induced to differentiate *in vitro* by removing the Leukemia Inhibitory Factor from the culture medium and through the formation of embryoid bodies (EBs), using the hanging drop method^29^,^56^ either in the presence or absence of ATO. ATO (Sigma, cat. n. 11099) was dissolved in 0.1N NaOH in milliQ water (Millipore) to a final concentration of 100 μM. This solution was added to the culture medium on day 0 to a final concentration of 0.1, 0.5 or 1.0 μM. As control samples, cells were cultured in the presence (CTR) of 0.01 N NaOH. ATO or NaOH were present in the culture medium throughout the 15 days differentiation.

For EBs formation, about seventy 20 μL droplets of culture medium containing 10^3^ mESCs were plated on the lid of p55 Petri dish. On day 3 of culture, the developing EBs were transferred on 0.1% agarose-coated tissue dishes (Corning) and from day 5, about 5–8 EBs were plated in single 1.9 cm^2^ well and cultivated up to 15 days. This procedure was repeated for three independent experiments. For each experiment, on day 4, 7 and 15 EBs were collected for real-time PCR analysis; on day 15 some EBs were video recorded for the analysis of their contractile properties, others were disaggregated for immunofluorescence analysis.

### RNA extraction

On day 4, 7 and 15 RNA was extracted using the GenElute Mammalian Total RNA Kit (Sigma, according to the manufacturer’s instruction) from about 250 EBs from CTR or 0.1, 0.5, 1.0 μM ATO-treated samples. Three independent experiments were performed and a total of about 3000 EBs analysed.

### Reverse transcription and quantitative Real-Time PCR

Reverse transcription and quantitative Real-Time PCR reactions were performed as previously described^55^,^57^. The sequences of specific primers used were reported in Table 1S. β-2-microglobulin gene expression^58^ was used for the normalization of the samples.

### Disaggregation of embryoid bodies

The immunofluorescence analysis was performed on EBs disaggregated at the end of the differentiation (day 15) process using a modified protocol (see supplementary materials) from Rebuzzini *et al*.^57^.

### Immunofluorescence

Immunostaining was performed using specific antibodies against the cardiac α-actinin (1:800, rabbit, Sigma–Aldrich), the cardiac isoform of troponin T (1:200, mouse, Thermo Scientific) and the connexin 43 (Cx43; 1:75, rabbit, Cell Signalling), as previously described^57^.

For each antibody and for each of the three independent experiments performed, one coverslip was examined and a total of about 400 cells were photographed and analysed.

### Protein extraction

Protein extraction from prenatal (E18) and newborn (P1) hearts of CD1 mice (Charles River), from CTR and ATO-treated (0.1, 0.5 and 1.0 μM) cells at day 15 of differentiation was performed as previously described^53^.

Animals were maintained in the department animal facility under temperature and humidity controlled conditions with a 12/12 hr dark/light cycle. All experiments were conducted in accordance to the protocol approved by our University and the European (n. 86/609/CEE) and Italian (n. 110 116/92, 8/94) legislation. The protocols were approved by the Ethical Committee of the University of Pavia (Protocol Number: 1; 2010).

### Western blotting

Five μg proteins were separated on 15% polyacrylamide gels and transferred on membranes (GE) overnight (4 °C, 40 V). Membranes were blocked with 1% BSA in PBS/0.05% Tween 20 for 30’ at room temperature. Cardiac myosin (Abcam, 1:1000 in PBS), troponin T (Thermo Scientific, 1:1000 in PBS), cardiac α-actinin (Sigma, 1:1500 in PBS) and gapdh (Abcam, 1:5000 in PBS) antibodies were incubated for 1 h and revealed with the appropriate secondary antibody (Sigma, 1:1000 in PBS) conjugated with horseradish peroxidase and detected using a commercial kit (BioRad). The intensity of the bands was quantified with Image J software (National Institute of Health, http://imagej.nih.gov/ij/).

### Contraction assays

On day 5 of culture, about 5–8 EBs were plated in 1.9 cm^2^ wells for a total of 20 wells. On a daily basis, from day 7 to 15 of differentiation, the number of beating EBs was counted^59^.

On a separate set of three independent experiments, about 20 EBs were plated onto 22 mm gelatin-coated Glass Bottom Dish (WillCo Wells), cultured up to day 15 and then transferred into the culture chamber of the Nikon BioStation IM at 37 °C and 5% CO_2_ for video recording. For each experiment, AVI videos of the beating syncytia were recorded, using the BioStation software, from ten randomly chosen CTR or ATO-treated cultures; the videos were then processed as previously described^39^,^57^.

For both assays, three sets of independent experiments were performed.

### Statistics

All data are presented as means ± standard deviation (SD), with the exception of the syncytium contractile properties that are expressed as mean ± 95% confidence interval for the differences between means. Data were analysed by the one-way ANOVA and by the *post hoc* LSD test. For immunofluorescence analysis, results are expressed as cell frequency (%) and analysed by the Chi-squared test.

## Additional Information

**How to cite this article**: Rebuzzini, P. *et al*. Arsenic trioxide alters the differentiation of mouse embryonic stem cell into cardiomyocytes. *Sci. Rep*. **5**, 14993; doi: 10.1038/srep14993 (2015).

## Supplementary Material

Supplementary Information

Supplementary Video S1

Supplementary Video S2

Supplementary Video S3

Supplementary Video S4

## Figures and Tables

**Table 1:** Ratio of sarcomeric proteins, referred to troponin T set at 1.

Samples	Troponin T	α-actinin	Myosin
*E18*	1	2.80 ± 0.01	1.52 ± 0.02
*P1*	1	2.92 ± 0.01	1.52 ± 0.02
*CTR*	1	2.91 ± 0.02	1.49 ± 0.02
*0.1* μ*M*	1	1.55 ± 0.03*	0.81 ± 0.01*
*0.5* μ*M*	1	0.71 ± 0.01*	0.24 ± 0.01*
*1.0* μ*M*	1	0.33 ± 0.01*	n.d.

^*^p < 0.001; n.d. not detectable.

## References

[b1] Chowdhury U. K. . Groundwater arsenic contamination in Bangladesh and West Bengal, India. Environ. Health. Perspect. 108, 393–397 (2000). 10811564 10.1289/ehp.00108393 PMC1638054

[b2] Dittmar J. . Arsenic in soil and irrigation water affects arsenic uptake by rice: complementary insights from field and pot studies. Environ. Sci. Technol. 44, 8842–8848 (2010). 21043519 10.1021/es101962d

[b3] States J. C. . Arsenic toxicology: translating between experimental models and human pathology. Environ. Health Perspect. 119, 1356–1363 (2011). 21684831 10.1289/ehp.1103441 PMC3230447

[b4] Naujokas M. F. . The broad scope of health effects from chronic arsenic exposure: update on a worldwide public health problem. Environ. Health Perspect. 121, 295–302 (2013). 23458756 10.1289/ehp.1205875 PMC3621177

[b5] Wang T. C., Jan K. Y., Wang A. S. & Gurr J. R. Trivalent arsenicals induce lipid peroxidation, protein carbonylation, and oxidative DNA damage in human urothelial cells. Mutat. Res. 615, 75–86 (2007). 17134727 10.1016/j.mrfmmm.2006.10.003

[b6] States J. C., Srivastava S., Chen Y. & Barchowsky A. Arsenic and cardiovascular disease. Toxicol. Sci. 107, 312–323 (2009). 19015167 10.1093/toxsci/kfn236 PMC2639760

[b7] Wade T. J. . Increased mortality associated with well-water arsenic exposure in Inner Mongolia, China. Int. J. Environ. Res. Public. Health 6, 1107–1123 (2009). 19440436 10.3390/ijerph6031107 PMC2672378

[b8] Moon K., Guallar E. & Navas-Acien A. Arsenic exposure and cardiovascular disease: an updated systematic review. Curr. Atheroscler. Rep. 14, 542–555 (2012). 22968315 10.1007/s11883-012-0280-x PMC3483370

[b9] Chen Y. & Karagas M. R. Arsenic and cardiovascular disease: new evidence from the United States. Ann. Intern. Med. 159, 713–714 (2013). 24061555 10.7326/0003-4819-159-10-201311190-00720 PMC4077583

[b10] Jomova K. . Arsenic: toxicity, oxidative stress and human disease. J. Appl. Toxicol. 31, 95–107 (2011). 21321970 10.1002/jat.1649

[b11] Smith A. H. & Steinmaus C. M. Arsenic in drinking water. BMJ 342 (2011). 10.1136/bmj.d2248 21546418

[b12] Chen C. J. . Increased prevalence of hypertension and long-term arsenic exposure. Hypertension 25, 53–60 (1995). 7843753

[b13] Rahman M. . Hypertension and arsenic exposure in Bangladesh. Hypertension 33, 74–78 (1999). 9931084 10.1161/01.hyp.33.1.74

[b14] Wang C. H. . Biological gradient between long-term arsenic exposure and carotid atherosclerosis. Circulation 105, 1804–1809 (2002). 11956123 10.1161/01.cir.0000015862.64816.b2

[b15] Lewis D. R., Southwick J. W., Ouellet-Hellstrom R., Rench J. & Calderon R. L. Drinking water arsenic in Utah: A cohort mortality study. Environ. Health Perspect. 107, 359–365 (1999). 10210691 10.1289/ehp.99107359 PMC1566417

[b16] Medrano M. A. . Arsenic in public water supplies and cardiovascular mortality in Spain. Environ. Res. 110, 448–454 (2010). 19880104 10.1016/j.envres.2009.10.002

[b17] Chen C. J., Chiou H. Y., Chiang M. H., Lin L. J. & Tai T. Y. Dose-response relationship between ischemic heart disease mortality and long-term arsenic exposure. Arterioscler. Thromb. Vasc. Biol. 16, 504–510 (1996). 8624771 10.1161/01.atv.16.4.504

[b18] Tseng C. H. . Long-term arsenic exposure and ischemic heart disease in arseniasis-hyperendemic villages in Taiwan. Toxicol. Lett. 137, 15–21 (2003). 12505429 10.1016/s0378-4274(02)00377-6

[b19] Quansah R. . Association of Arsenic with Adverse Pregnancy Outcomes-Infant Mortality: A Systematic Review and Meta-Analysis. Environ. Health Perspect. 27 (2015). 10.1289/ehp.1307894 PMC4421764 25626053

[b20] Chen Y. . Arsenic exposure from drinking water and mortality from cardiovascular disease in Bangladesh: prospective cohort study. BMJ 342 (2011). 10.1136/bmj.d2431 PMC3088786 21546419

[b21] Raghu K. G. . Evaluation of adverse cardiac effects induced by arsenic trioxide, a potent anti-APL drug. J. Environ. Pathol. Toxicol. Oncol. 28, 241–252 (2009). 19888912 10.1615/jenvironpatholtoxicoloncol.v28.i3.60

[b22] Shen Z. X. . All-trans retinoic acid/As2O3 combination yields a high quality remission and survival in newly diagnosed acute promyelocytic leukemia. Proc. Natl. Acad. Sci. USA 101, 5328–5335 (2004). 15044693 10.1073/pnas.0400053101 PMC397380

[b23] Wang Z. Y. & Chen Z. Acute promyelocytic leukemia: from highly fatal to highly curable. Blood. 111, 2505–2515 (2008). 18299451 10.1182/blood-2007-07-102798

[b24] Platanias L. C. Biological responses to arsenic compounds. J. Biol. Chem. 284, 18583–18587 (2009). 19363033 10.1074/jbc.R900003200 PMC2707240

[b25] Zhao X. . Arsenic trioxide-induced apoptosis in H9c2 cardiomyocytes: implications in cardiotoxicity. Basic. Clin. Pharmacol. Toxicol. 102, 419–425 (2008). 18346055 10.1111/j.1742-7843.2007.00150.x

[b26] Sumi D., Sasaki T., Miyataka H. & Himeno S. Rat H9c2 cardiac myocytes are sensitive to arsenite due to a modest activation of transcription factor Nrf2. Arch. Toxicol 85, 1509–1516 (2011). 21465251 10.1007/s00204-011-0700-7

[b27] Stummann T. C., Hareng L. & Bremer S. Embryotoxicity hazard assessment of cadmium and arsenic compounds using embryonic stem cells. Toxicology 252, 118–122 (2008). 18775467 10.1016/j.tox.2008.08.001

[b28] Wang Q. Q. . Effect of arsenic compounds on the *in vitro* differentiation of mouse embryonic stem cells into cardiomyocytes. Chem. Res. Toxicol. 28, 351–353 (2015). 25166275 10.1021/tx500286t

[b29] Boheler K. R. . Differentiation of pluripotent embryonic stem cells into cardiomyocytes. Circ. Res. 91, 189–201 (2002). 12169644 10.1161/01.res.0000027865.61704.32

[b30] Mercola M., Ruiz-Lozano P. & Schneider M. D. Cardiac muscle regeneration: lessons from development. Genes Dev. 25, 299–309 (2011). 21325131 10.1101/gad.2018411 PMC3042154

[b31] Naranmandura H., Suzuki N. & Suzuki K. T. Trivalent arsenicals are bound to proteins during reductive methylation. Chem. Res. Toxicol. 19, 1010–1018 (2006). 16918239 10.1021/tx060053f

[b32] Cullen W. R. Chemical mechanism of arsenic biomethylation. Chem. Res. Toxicol. 27, 457–461 (2014). 24517124 10.1021/tx400441h

[b33] Wobus A. M., Guan K., Yang H. T. & Boheler K. R. Embryonic stem cells as a model to study cardiac, skeletal muscle, and vascular smooth muscle cell differentiation. Methods Mol. Biol. 185, 127–156 (2002). 11768985 10.1385/1-59259-241-4:127

[b34] Heikinheimo M., Scandrett J. M. & Wilson D. B. Localization of transcription factor GATA-4 to regions of the mouse embryo involved in cardiac development. Dev. Biol. 164, 361–373 (1994). 8045339 10.1006/dbio.1994.1206

[b35] Ip H. S. . The GATA-4 transcription factor transactivates the cardiac muscle-specific troponin C promoter-enhancer in nonmuscle cells. Mol. Cell Biol. 14, 7517–7526 (1994). 7935467 10.1128/mcb.14.11.7517-7526.1994 PMC359288

[b36] Monzen K. . Bone morphogenetic proteins induce cardiomyocyte differentiation through the mitogen-activated protein kinase TAK1 and cardiac transcription factors Csx/Nkx-2.5 and GATA-4. Mol. Cell Biol. 19, 7096–7105 (1999). 10490646 10.1128/mcb.19.10.7096 PMC84704

[b37] Kehat I. . Human embryonic stem cells can differentiate into myocytes with structural and functional properties of cardiomyocytes. J. Clin. Invest. 108, 407–414 (2001). 11489934 10.1172/JCI12131 PMC209357

[b38] Xu C., Police, S., Rao, N. & Carpenter, M.K. Characterization and enrichment of cardiomyocytes derived from human embryonic stem cells. Circ. Res. 91, 501–508 (2002). 12242268 10.1161/01.res.0000035254.80718.91

[b39] Fassina L. . Video evaluation of the kinematics and dynamics of the beating cardiac syncytium: an alternative to the Langendorff method. Int. J. Artif. Organs 34, 546–558 (2011). 21786253 10.5301/IJAO.2011.8510

[b40] Boheler K. R., Crider D. G., Tarasova Y. & Maltsev V. A. Cardiomyocytes derived from embryonic stem cells. Methods Mol. Med. 108, 417–435 (2005). 16028698 10.1385/1-59259-850-1:417

[b41] van Halem D., Bakker S. A., Amy G. L. & van Dijk J. C. Arsenic in drinking water: a worldwide water quality concern for water supply compagnie. Drink Water Eng. Sci. 2, 29–34 (2009).

[b42] Durocher D., Charron F., Warren R., Schwartz R. J. & Nemer M. The cardiac transcription factors Nkx2-5 and GATA-4 are mutual cofactors. EMBO J. 15, 5687–5696 (1997). 9312027 10.1093/emboj/16.18.5687 PMC1170200

[b43] Lien C. L. . Control of early cardiac-specific transcription of Nkx2-5 by a GATA-dependent enhancer. Development 126, 75–84 (1999). 9834187 10.1242/dev.126.1.75

[b44] Riazi A. M. . NKX2-5 regulates the expression of beta-catenin and GATA4 in ventricular myocytes. PLoS One. 4, e5698 (2009). 19479054 10.1371/journal.pone.0005698 PMC2684637

[b45] Verma V., Purnamawati K., Manasi & Shim W. Steering signal transduction pathway towards cardiac lineage from human pluripotent stem cells: a review. Cell Signal. 25, 1096–107 (2013). 23415770 10.1016/j.cellsig.2013.01.027

[b46] Hong G. M. & Bain L. J. Arsenic exposure inhibits myogenesis and neurogenesis in P19 stem cells through repression of the β-catenin signaling pathway. Toxicol. Sci. 129, 146–156 (2012). 22641621 10.1093/toxsci/kfs186 PMC3499077

[b47] Yen Y. P. . Arsenic inhibits myogenic differentiation and muscle regeneration. Environ. Health Perspect. 118, 949–956 (2010). 20299303 10.1289/ehp.0901525 PMC2920914

[b48] Steffens A. A., Hong G. M. & Bain L. J. Sodium arsenite delays the differentiation of C2C12 mouse myoblast cells and alters methylation patterns on the transcription factor myogenin. Toxicol. Appl. Pharmacol. 250, 154–161 (2011). 20965206 10.1016/j.taap.2010.10.006 PMC3014457

[b49] Sumi D., Abe K. & Himeno S. Arsenite retards the cardiac differentiation of rat cardiac myoblast H9c2 cells. Biochem. Biophys. Res. Commun. 436, 175–179 (2013). 23727579 10.1016/j.bbrc.2013.05.069

[b50] Thompson B. R. & Metzger J. M. Cell biology of sarcomeric protein engineering: disease modeling and therapeutic potential. Anat. Rec. (Hoboken) 297, 1663–1669 (2014). 25125179 10.1002/ar.22966 PMC4133785

[b51] Chou Y. . Endothelial gap junctions are down-regulated by arsenic trioxide. Eur. J. Pharmacol. 569, 29–36 (2007). 17559834 10.1016/j.ejphar.2007.05.011

[b52] Vink M. J. . Alterations of intercellular communication in neonatal cardiac myocytes from connexin43 null mice. Cardiovasc. Res. 62, 397–406 (2004). 15094359 10.1016/j.cardiores.2004.01.015

[b53] Mo J. . Altered gene expression by low-dose arsenic exposure in humans and cultured cardiomyocytes: assessment by real-time PCR arrays. Int. J. Environ. Res. Public Health 8, 2090–2108 (2011). 21776218 10.3390/ijerph8062090 PMC3138013

[b54] Farzan S. F., Karagas M. R. & Chen Y. In utero and early life arsenic exposure in relation to long-term health and disease. Toxicol. Appl. Pharmacol. 272, 384–390 (2013). 23859881 10.1016/j.taap.2013.06.030 PMC3783578

[b55] Rebuzzini P. . Mouse embryonic stem cells that survive γ-rays exposure maintain pluripotent differentiation potential and genome stability. J. Cell. Physiol. 227, 1242–1249 (2012). 21732352 10.1002/jcp.22908

[b56] Neri T. . The differentiation of cardiomyocytes from mouse embryonic stem cells is altered by dioxin. Toxicol. Lett. 202, 226–236 (2011). 21354282 10.1016/j.toxlet.2011.02.008

[b57] Rebuzzini P. . Mouse embryonic stem cells irradiated with γ-rays differentiate into cardiomyocytes but with altered contractile properties. Mutat. Res. 756, 37–45 (2013). 23792212 10.1016/j.mrgentox.2013.06.007

[b58] Banda M., Bommineni A., Thomas R. A., Luckinbill L. S. & Tucker J. D. Evaluation and validation of housekeeping genes in response to ionizing radiation and chemical exposure for normalizing RNA expression in real-time PCR. Mutat. Res. 649, 126–134 (2008). 17904413 10.1016/j.mrgentox.2007.08.005

[b59] zur Nieden N. I., Kempka G. & Ahr H. J. Molecular multiple endpoint embryonic stem cell test–a possible approach to test for the teratogenic potential of compounds. Toxicol. Appl. Pharmacol. 194, 257–269 (2004). 14761682 10.1016/j.taap.2003.09.019

