# Supplementary material for: Arsenic trioxide alters the differentiation of mouse embryonic stem cell into cardiomyocytes
Source: Sci Rep. 2015 Oct 8;5:14993. doi: 10.1038/srep14993 (PMC4597215; doi:10.1038/srep14993)
Supplement: Supplementary Information [file srep14993-s1.pdf]

Arsenic trioxide alters the differentiation of mouse embryonic stem cell into cardiomyocytes

Paola Rebuzzini[a,d], Elisa Cebral[b], Lorenzo Fassina[c,d], Carlo Alberto Redi[a,d,e], Maurizio Zuccotti[f,*] and Silvia Garagna[a,d,*]

[a]Laboratorio di Biologia dello Sviluppo, Dipartimento di Biologia e Biotecnologie 'Lazzaro Spallanzani', Università degli Studi di Pavia, Pavia, Italy;
[b]Laboratorio de Reproducción y Fisiopatología Materno-Embrionaria, Instituto de Fisiología, Biología Molecular y Neurociencias, Departamento de Biodiversidad y Biología Experimental, Facultad de Ciencias Exactas y Naturales, Universidad de Buenos Aires, Buenos Aires, Argentina;
[c]Dipartimento di Ingegneria Industriale e dell'Informazione, Università degli Studi di Pavia, Pavia, Italy;
[d]Center for Health Technologies (CHT), Via Ferrata 1, University of Pavia, Italy;
[e]Centro Ricerche di Medicina Rigenerativa, Fondazione IRCCS Policlinico San Matteo, Pavia, Italy;
[f]Unita' di Anatomia, Istologia ed Embriologia, Dipartimento di Scienze Biomediche, Biotecnologiche e Traslazionali, Università degli Studi di Parma, Parma, Italy.

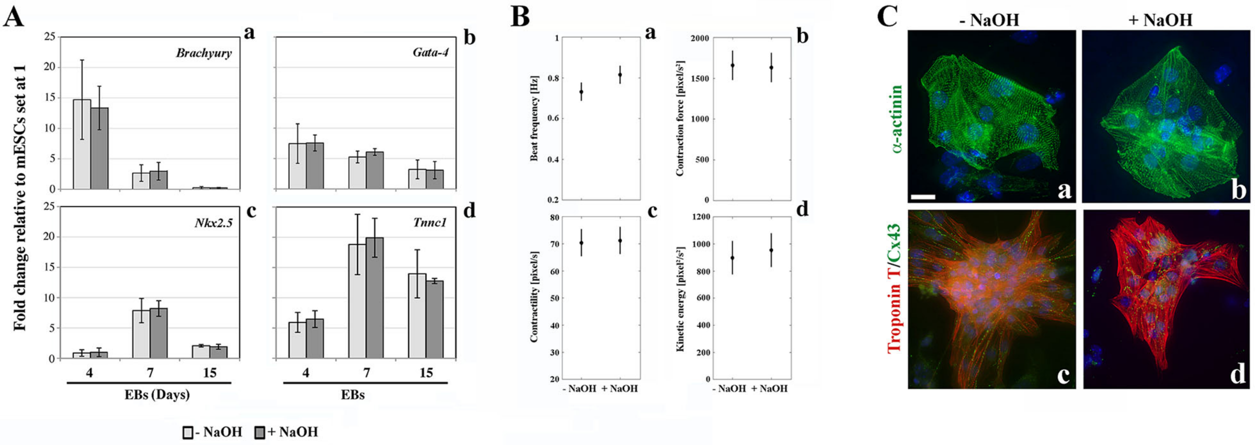

A
a
b
c
d
Brachyury
Gata-4
Nkx2.5
Tnnc1
Fold change relative to mESCs set at 1
EBs (Days)
EBs
- NaOH
+ NaOH
B
Beat frequency [Hz]
Contraction force [pixel/s²]
Contractility [pixel/s]
Kinetic energy [pixel²/s²]
C
α-actinin
Troponin T/Cx43

Figure 1S. Cardiomyocyte differentiation in the absence or presence of 0.01 N NaOH. **A)** Expression profile of genes that mark mesoderm (**a**, *Brachyury*), cardiac mesoderm (**b**, *Gata-4;* **c**, *Nkx2.5*) and cardiac cells (**d**, *Tnnc1*) throughout the differentiation process. Values are expressed as mean±standard deviation; **B)** Contractile properties of beating syncytia on day 15: **a)** Beat frequency [Hz]; **b)** Contraction force [pixel/s^2]; **c)** Contractility [pixel/s]; **d)** Kinetic energy [$pixel^2/s^2$]. Horizontal bars represent the 95% confidence interval for the differences between means according to the Least Significant Difference statistical test. **C)** Immunofluorescence localisation of cardiac α-actinin and of cardiac troponin T (red) and Connexin 43 (green) proteins in cardiomyocytes on day 15. Bar, 20 μm.

Three independent sets of experiments were performed.

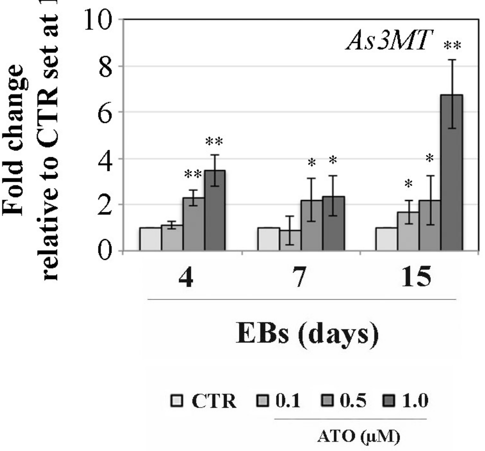

Fold change
relative to CTR set at 1
10
8
6
4
2
0
As3MT
**
**
**
*
*
*
*
**
4
7
15
EBs (days)
CTR
0.1
0.5
1.0
ATO (μM)

Figure 2S. Expression profile of *As3MT* in CTR and ATO-exposed EBs. Values are expressed as mean±standard deviation. Three independent sets of this experiment were performed. *p<0.05; **p<0.001.

Methods

Disaggregation of embryoid bodies

Briefly, for each experiment, about 100 EBs were mechanically detached and centrifuged at 500 rpm for 5 min and resuspended in 1 ml of "low Ca^{2+}-medium" (120 mM NaCl, 5.4 mM KCl, 5 mM sodium pyruvate, 20 mM glucose, 20 mM taurine, 10 mM HEPES) for 15 min at room temperature. Then, cells were incubated in "low Ca^{2+}-medium" supplemented with 1 mg/ml collagenase and 30 mM $CaCl_2$ for 30 min at 37°C. After centrifugation at 500 rpm for 5 min, cells were resuspended in complete culture medium and about 50,000 cells seeded on each 0.1% gelatin-coated coverslip. After 24 h, all cells were washed twice with PBS, fixed in 4% cold paraformaldehyde/PBS. Slides were kept at 4°C until usage.

Table 1S. Oligonucleotides used for Real Time PCR amplification.

Gene	Primer forward	Primer Reverse	Amplicon length (bp)
Brachyury	5' CTCTAAGGAACCACCGGTCA 3'	5' AGCATGGACAGACAAGCAGA 3'	100
Gata-4	5' AGTTGTGCAGCTAATGCCACT 3'	5' CTGCTTTGGTAGCAGGTTTTG 3'	100
Nkx2.5	5'GATGGGAAAGCTCCCACTATG 3'	5'GAGACACCAGGCTACGTCAATA 3'	110
Myh6	5' TCACTGCGGAAACTGAAAACG 3'	5' ATGGCCATGTCCTCGATCTTG 3'	100
Actn2	5' AACCTGGCCATGGAAATAGCA 3'	5' TTCATCGGGTTTGGGAGTGTT 3'	90
Tnnc1	5' CAGCAAAGGGAAGTCTGAGG 3'	5' TGCAGCATCATCTTCAGCTC 3'	102
Tnnt2	5' GAAGTTCGACCTGCAGGAAA 3'	5' TTCCCACGAGTTTTGGAGAC 3'	102
Tnni3	5' GACTTATGCCGACAGCTTCAC 3'	5' GGTCAGATCTGCAATCTCAGTG 3'	105
As3MT	5' AAAACAAGGAGCTCGAAGGGG 3'	5' TTTCGGCTGGCTCTGTCTTAG 3'	93
β2m	5' GAATTCACCCCCACTGAGACT 3'	5' TGCTTGATCACATGTCTCGAT 3'	103
